# Association between glycated albumin and sudden death in patients undergoing hemodialysis

**DOI:** 10.1007/s10157-024-02475-w

**Published:** 2024-03-04

**Authors:** Yoshiki Kaizu, Masaharu Nagata, Shinako Kaizu, Yueling Qie, Kazo Kaizu, Shigeru Tanaka, Toshiaki Nakano, Takanari Kitazono

**Affiliations:** 1 Kitakyusyujinzo Clinic, Fukuoka, Japan; 2 Shin-Eikai Hospital, 14-11 Benten-Cho, Kokurakita-Ku, Kitakyushu-City, Fukuoka, 803-0856 Japan; 3 Shinkitakyusyujinzo Clinic, Fukuoka, Japan; 4 Department of Medicine and Clinical Science, Graduate School of Medical Sciences, Kyushu University, Fukuoka, Japan

**Keywords:** Glycated albumin, Sudden death, Hemodialysis, Diabetes

## Abstract

**Background:**

The frequency of sudden death and its risk factors in patients undergoing hemodialysis are unknown. This study was performed to examine the association between glycated albumin (GA) and sudden death in Japanese patients undergoing hemodialysis.

**Methods:**

In total, 260 patients undergoing hemodialysis aged ≥18 years were retrospectively followed for a mean of 4.6 years. The patients’ serum GA levels were divided into tertiles, and the patients’ sex, age, albumin level, C-reactive protein (CRP) level, and cardiothoracic ratio (CTR) were selected as adjustment factors. A logistic regression model was used to calculate the odds ratio (OR) for the occurrence of sudden death by GA level.

**Results:**

Ninety-one patients died during follow-up. Of the 91 deaths, 23 (25.2%) were defined as sudden deaths. Compared with non-sudden death cases, sudden death cases were significantly younger (*p* = 0.002) and had a higher proportion of men (*p* = 0.03), a higher proportion of diabetes (*p* = 0.008), and higher GA levels (*p* = 0.023). Compared with patients with the lowest GA levels (<15.2%), those with the highest GA levels (≥18.5%) had a sex- and age-adjusted OR for sudden death of 5.40 [95% confidence interval (CI): 1.35–21.85]. After adjusting for the albumin level, CRP level, and CTR in addition to sex and age, the OR for sudden death of patients with the highest GA levels increased to 6.80 (95%CI: 1.64–28.08); the relationship did not change.

**Conclusion:**

Serum GA levels were significantly associated with sudden death in patients undergoing hemodialysis.

**Supplementary Information:**

The online version contains supplementary material available at 10.1007/s10157-024-02475-w.

## Introduction

Sudden death is typically defined as death from intrinsic causes occurring within 24 h of onset, and chronic kidney disease is reportedly an independent risk factor for sudden death [1]. The incidence of sudden cardiac death in patients undergoing hemodialysis is extremely high (25–130 times higher than that in the general population), and sudden death reportedly accounts for 13–28% of all deaths [2–4].

Risk factors for sudden death in patients undergoing hemodialysis include male sex, advanced age, cardiovascular disease, atrial fibrillation, a higher cardiothoracic ratio (CTR), a higher C-reactive protein (CRP) level, predialytic hyperphosphatemia, and predialytic hyperkalemia [2, 5]. Patients with diabetes mellitus undergoing hemodialysis are at particularly high risk of sudden death [2, 5].

HemoglobinA1c(HbA1c) and glycated albumin (GA) are used as indices for diabetes management in patients undergoing hemodialysis, but GA is more accurate than HbA1c in assessing glycemic control during hemodialysis [6–8]. In addition to being an indicator of glycemic control, GA has been shown to be significantly related to life expectancy [9]. GA may be an important index with which to evaluate the prognosis of patients undergoing hemodialysis, but the effects of GA on sudden death have not been reported to date. Therefore, the current study was performed to examine the relationship between GA and sudden death in patients undergoing hemodialysis.

## Materials and methods

### Patients

This study involved 260 patients (165 men, 95 women) undergoing maintenance hemodialysis from January 2016 to the end of December 2017. The patients’ mean age was 68 ± 14 years, their mean time on dialysis was 7.6 years, and they were followed until the end of December 2021 (mean follow-up: 4.6 years).

### Methodology

Sudden death was defined a witnessed death within 24 h of onset of acute symptoms, and witnessed unexpected death within the interval between dialysis sessions and excluded trauma, asphyxia, and suicide. This definition has been used in previous studies [2, 5]. Sudden deaths that occurred at other hospitals were confirmed by phoning the medical facility and consulting medical records, and the direct causes of sudden death were examined to the greatest extent possible.

### Clinical course parameters

The following information was collected from the medical records to serve as clinical parameters: age, sex, time on dialysis, body mass index, presence or absence of diabetes mellitus, ischemic heart disease, cerebrovascular disease, peripheral arterial disease, or valvular heart disease, treatment with anti-platelet agents, antihypertensive agents (angiotensin-converting enzyme inhibitors/angiotensin receptor blockers/calcium antagonists), iron therapy (oral/intravenous), an active form of vitamin D (oral/intravenous), or erythropoietin, predialysis systolic and diastolic blood pressure, levels of hemoglobin, serum albumin, phosphorus, corrected calcium, intact parathyroid hormone, magnesium, potassium, creatinine, urea nitrogen, and ferritin, protein catabolic rate, Kt/V, GA level, and cardio-thoracic ratio (CTR) according to chest X-rays. Blood tests were performed 2 days before dialysis at the start of the week and at least 3 months after the start of dialysis.

Baseline characteristics are expressed as mean ± standard deviation for continuous variables and as percentage for categorical variables. Significant differences between patients with and without sudden death were evaluated using Student’s t test for continuous variables and the chi-square test for categorical variables.

The patients’ GA levels were divided into tertiles (<15.2, 15.2–18.5, and ≥18.5%). Multiple variables in the relationship between the assessed GA levels and sudden death were adjusted for in a logistic regression model. Sex, age, albumin level, CRP level, and the CTR were selected as adjustment factors, and the odds ratio (OR) of sudden death was calculated by GA level. Statistical calculations were performed using JMP version 16 (SAS Institute Inc., Cary, NC, USA).

## Results

The characteristics of all of the population in this study and comparison of the characteristics of sudden death case and non-sudden death cases are shown in Table 1. In total, 91 deaths occurred during follow-up. Of these 91 deaths, 23 (25.2%) were defined as sudden deaths. Compared with patients without sudden death, those with sudden death were significantly younger (*p* = 0.002), had a higher proportion of men (*p* = 0.03), had a higher proportion of diabetes (*p* = 0.008), and had higher GA levels (*p* = 0.023) (Table 1).

**Table 1:** Baseline characteristics of the study patients, stratified by sudden death versus non-sudden death

	All patients (*n* = 260)	All death (*n* = 91)		*p* for trend^*^
		Sudden death (*n* = 23)	Non-sudden death (*n* = 68)	
Age (years)	68 (14)	70 (2)	78 (1)	0.002
Male (%)	63	87	62	0.03
Hemodialysis vintage (years)	7.6	7.2	7.0	0.94
Dialysis session length (h)	4.3 (0.6)	4.1 (0.1)	4.1 (0.1)	0.89
Diabetes (%)	33.0	40.5	14.8	0.008
History of IHD (%)	28.4	34.7	34.3	0.57
History of PAD (%)	19.2	21.7	34.3	0.24
Systoloc blood pressure (mmHg)	138 (21)	133 (5)	137 (3)	0.47
Diastolic blood pressure (mmHg)	74 (14)	72 (3)	69 (2)	0.36
Body mass index (kg/m^2^)	21.0 (3.4)	21.0 (0.7)	19.0 (0.4)	0.16
Cardiothoratic ratio (%)	50.0 (4.7)	52.0 (1.1)	51.0 (0.6)	0.57
Kt/V for urea	1.54 (0.28)	1.51 (0.05)	1.46 (0.03)	0.43
Hemoglobin (g/dL)	11.3 (1.3)	11.3 (0.3)	11.3 (0.1)	0.36
Serum alubumin (g/dL)	3.9 (0.4)	3.7 (0.1)	3.8 (0.1)	0.55
Serum potassium (mEq/L)	4.8 (0.7)	4.8 (0.2)	4.7 (0.1)	0.35
Serum corrected calcium (mg/dL)	9 (0.6)	9.1 (0.1)	8.9 (0.1)	0.15
Serum phosphorus (mg/dL)	5.2 (1.3)	5.2 (0.3)	5.0 (0.2)	0.42
Serum CRP (mg/dL)	0.6 (1.2)	1.3 (0.3)	1.0 (0.2)	0.32
Serum Glycated albumin (g/dL)	17.7 (4.8)	22.0 (1.1)	17.3 (0.3)	0.023
Serum cholesterol (mg/dL)	156 (36)	150 (8)	149 (5)	0.91
Serum ferritin (ng/mL)	115 (171)	169 (37)	165 (11)	0.90
Use of ESA (%)	85.5	95.5	83.8	0.15
Use of phosphate-binders (%)	78.6	74.0	79.2	0.57
Use of anti-platelet agents (%)	46.1	52.2	45.5	0.54
Use of iron supplementation (%)	10.8	13.0	9.0	0.58

Data are presented as mean (standard deviation) for continuous variables and as percentage for categorical variables

*IHD* ischemic heart disease, *PAD* peripheral arterial disease, *CRP* C-reactive protein

^*^Sudden death versus non-sudden death (*p* < 0.05)

The GA levels were divided into tertiles (T1: GA <15.2%, T2: GA 15.2–18.5%, and T3: GA ≥18.5%), and the characteristics of the patients in each tertile are compared in Table 2. Compared with the patients in T1 and T2, the patients in T3 tended to be older, tended to have been undergoing dialysis for less time, were more likely to have diabetes, had a higher proportion of peripheral arterial disease, and tended to have lower diastolic blood pressure, higher CRP levels, and lower total cholesterol levels.

**Table 2:** Patients’ characteristics according to serum glycated albumin tertiles

	Serum glycated albumin (%)			
	T1 (<15.2) (*n* = 87)	T2 (15.2–18.5) (*n* = 86)	T3 (≥18.5) (*n* = 87)	*p* for trend
Age (years)	61 (1.3)	72 (1.3)	72 (1.3)	<0.001
Male (%)	59.7	66.3	64.4	0.65
Hemodialysis vintage (years)	10.5	6.2	6.0	<0.001
Diabetes (%)	4.6	22.1	72.4	<0.001
Body mass index (kg/m^2^)	21.0 (0.4)	21.0 (0.4)	20.0 (0.4)	0.37
History of IHD (%)	21.8	27.9	35.6	0.12
History of PAD (%)	6.9	15.1	35.6	<0.001
Systoloc blood pressure (mmHg)	133 (16)	134 (21)	141 (24)	0.12
Diastolic blood pressure (mmHg)	80 (14)	72 (14)	71 (14)	<0.001
Cardiothoratic ratio (%)	49.3 (0.5)	49.9 (0.5)	50.0 (0.5)	0.56
Kt/V for urea	1.6 (0.3)	1.5 (0.2)	1.5 (0.3)	<0.001
Hemoglobin (g/dL)	11.3 (1.2)	11.3 (1.3)	11.3 (1.4)	0.88
Serum alubumin (g/dL)	4.0 (0.3)	3.8 (0.4)	3.9 (0.4)	0.011
Serum potassium (mEq/L)	5.0 (0.7)	4.7 (0.8)	4.8 (0.7)	0.003
Serum corrected calcium (mg/dL)	9.0 (0.6)	8.9 (0.6)	8.9 (0.6)	0.27
Serum phosphorus (mg/dL)	5.5 (1.1)	5.0 (1.3)	5.2 (1.3)	0.038
Serum CRP (mg/dL)	0.46 (1.20)	0.32 (0.50)	0.88 (1.60)	0.007
Serum cholesterol (mg/dL)	164 (39)	153 (33)	151 (35)	0.032
Serum ferritin (ng/mL)	131 (18)	165 (18)	199 (18)	0.033
Use of ESA (%)	72.4	88.1	94.2	<0.001
Use of phosphate-binders (%)	93.0	70.0	73.3	<0.001
Use of anti-platelet agents (%)	28.0	57.0	53.5	<0.001
Use of iron supplementation (%)	13.0	7.0	13.0	0.34

Data are presented as mean (standard deviation) for continuous variables and as percentage for categorical measures

*IHD* ischemic heart disease, *PAD* peripheral arterial disease, *CRP* C-reactive protein

**Fig. 1:**
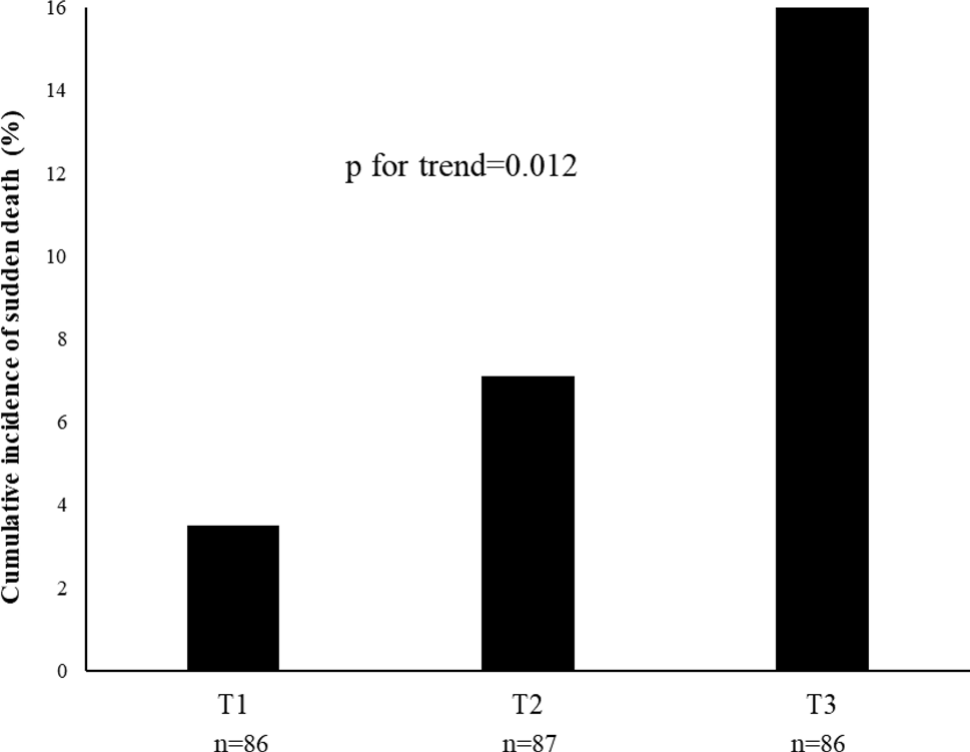
The cumulative incidence of sudden death by glycated albumin level

The cumulative incidence of sudden death by GA level is shown in Fig. 1. The incidence of sudden death increased linearly: 3.5% in T1, 7.1% in T2, and 16.1% in T3 (*p* for trend = 0.012).

**Fig. 2:**
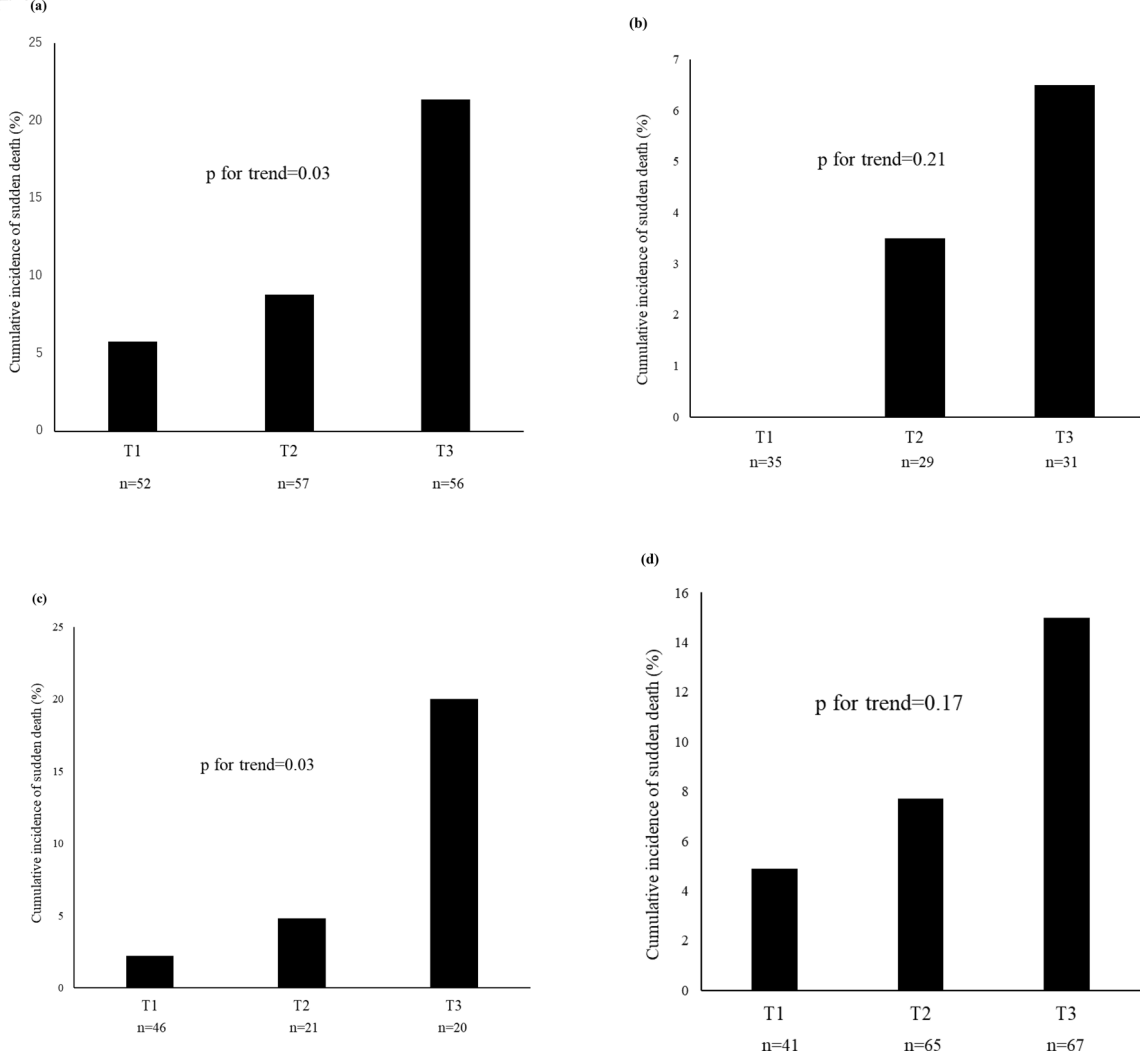
The cumulative incidence of sudden death for sex and age by glycated albumin level **a** male, **b** female, **c** <65 years group, and **d** ≥65 years group

Next, subgroup analyses were performed by sex and age (≥65 vs. <65 years). A significant increase in the cumulative incidence of sudden death was noted in men: 5.8% in T1, 8.8% in T2, and 21.4% in T3 (*p* for trend = 0.03) (Fig. 2a). A linear increase was also noted in women: 0.0% in T1, 3.5% in T2, and 6.5% in T3; however, the difference was not statistically significant (*p* for trend = 0.21) (Fig. 2b).

A significant increase in the cumulative incidence of sudden death was noted in patients aged <65 years: 2.2% in T1, 4.8% in T2, and 20.0% in T3 (*p* for trend = 0.03) (Fig. 2c). A gradual increase in the cumulative incidence of sudden death was noted in patients aged ≥65 years, but the difference was not statistically significant (Fig. 2d).

The OR of sudden death by GA level is shown in Table 3. The sex- and age-adjusted OR of sudden death in T3 (≥18.5%) was 5.4 [confidence interval (CI): 1.35–21.85] compared with T1 (<15.2%). After adjusting for the serum albumin level, serum CRP level, and CTR in addition to sex and age, the OR of sudden death in T3 was 6.8 (95% CI: 1.64–28.08).

**Table 3:** Odds ratio for sudden death according to serum glycated albumin

		Number of events	Number of subjects	Model 1^a^		Model 2^b^	
				OR (95% CI)	*p* value	OR (95% CI)	*p* value
*Glycated albumin*							
T1	<15.2	3	87	1.00 (reference)		1.00 (reference)	–
T2	15.2–18.5	6	86	2.02 (0.45–9.04)	0.36	1.90 (0.40–9.94)	0.40
T3	≥18.5	14	87	5.40 (1.35–21.85)	0.002	6.80 (1.64–28.08)	0.008

*OR* odds ratio, *CI* confidence interval

^a^Model 1: age and sex

^b^Model 2:age, sex, serum albumin level, serum C-reactive protein (CRP) level and cardiothoracic ratio (CTR)

The subgroup analysis of patients aged ≥65 and <65 years is shown in Table 4. After adjusting for sex and age, the OR of sudden death for ≥65-year-old patients in T3 was 3.24 (95% CI: 0.64–16.25) compared with that in T1. After adjusting for the serum albumin level, serum CRP level, and CTR in addition to sex and age, the OR of sudden death in T3 was 4.09 (95% CI: 0.79–21.18). After adjusting for sex and age, the OR of sudden death for <65-year-old patients in T3 was 9.24 (95% CI: 0.75–114.50) compared with that in T1. After adjusting for the serum albumin level, serum CRP level, and CTR in addition to sex and age, the OR of sudden death for patients in T3 was 0.16 (95% CI: 0.00–66.50).

**Table 4:** Odds ratio for sudden death according to serum glycated albumin of patients aged ≥65 and <65 years

Glycated albumin		Number of events	Number of subjects	Model 1^a^		Model 2^b^	
				OR (95% CI)	*p* value	OR (95% CI)	*p* value
*≥65 years*							
T1	<15.2	2	41	1.00 (reference)		1.00 (reference)	–
T2	15.2–18.5	5	65	1.53 (0.27–8.65)	0.63	1.45 (0.23–9.21)	0.69
T3	≥18.5	10	67	3.24 (0.64–16.25)	0.15	4.09 (0.79–21.18)	0.09
*<65 years*							
T1	<15.2	1	46	1.00 (reference)		1.00 (reference)	–
T2	15.2–18.5	1	21	1.81 (0.10–32.77)	0.68	0.47 (0.00–25.53)	0.71
T3	≥18.5	4	20	9.24 (0.75–114.50)	0.08	0.16 (0.00–66.5)	0.55

*OR* odds ratio, *CI* confidence interval

^a^Model 1: age and sex

^b^Model 2: age, sex, serum albumin level, serum C-reactive protein (CRP) level and cardiothoracic ratio (CTR)

The subgroup analysis by sex is shown in Table 5. After adjusting for sex and age, the OR of sudden death for men in T3 was 4.52 (95% CI: 1.08–19.00) compared with that in T1. After adjusting for the serum albumin level, serum CRP level, and CTR in addition to sex and age, the OR of sudden death for men in T3 was 5.96 (95% CI: 1.39–25.50). Sudden death did not occur among women in T1, so the OR could not be calculated.

**Table 5:** Odds ratio for sudden death according to serum glycated albumin by sex

Glycated albumin		Number of events	Number of subjects	Model 1^a^		Model 2^b^	
				OR (95% CI)	*p* value	OR (95% CI)	*p* value
*Male*							
T1	<15.2	3	53	1.00 (reference)		1.00 (reference)	–
T2	15.2–18.5	5	56	1.62 (0.34–7.70)	0.54	1.67 (0.31–8.88)	0.55
T3	≥18.5	12	56	4.52 (1.08–19.0)	0.04	5.96 (1.39–25.50)	0.02
*Female*							
T1	<15.2	0	35	1.00 (reference)		1.00 (reference)	–
T2	15.2–18.5	1	39	–		–	
T3	≥18.5	2	31	–		–	

*OR* odds ratio, *CI* confidence interval

^a^Model 1: age and sex

^b^Model 2: age, sex, serum albumin level, serum C-reactive protein (CRP) level and cardiothoracic ratio (CTR)

## Discussion

This retrospective follow-up study revealed the frequency of sudden death in 260 patients undergoing hemodialysis. During the 4.6-year follow-up, sudden death accounted for 25.2% of all deaths. This frequency did not differ substantially from the rate of sudden death in patients undergoing hemodialysis in previous studies [2, 5].

Diabetes is a reported risk factor for sudden death in patients undergoing hemodialysis [2, 5]. The presence of systemic vascular atherosclerosis due to diabetes and cardiovascular disease due to chronic inflammation may be involved in the occurrence of sudden death, but no studies have examined the effects of GA on sudden death in patients undergoing hemodialysis.

HbA1c is an index reflecting the glycemic status, and it is well known to be significantly associated with the progression of cardiovascular disease. Like HbA1c, GA is considered to be a clinically useful marker to evaluate the risk of cardiovascular disease. GA is the product of the glycation of serum albumin, and it reflects the glycemic status of the previous 2–4 weeks. Because GA is not affected by the erythrocyte lifespan or the administration of erythropoiesis-stimulating agents, it is considered a more useful indicator of glycemic control than HbA1c in patients with chronic kidney disease, particularly those undergoing dialysis. In the current study, GA was a significant risk factor for sudden death.

Chen et al. [10] measured the GA level multiple times in patients with diabetes undergoing hemodialysis, and they divided the GA levels into quartiles and examined survival. Patients with the highest GA levels had a 42% higher mortality rate than patients with the lowest GA levels [10]. Similarly, Lu et al. [11] reported that patients undergoing hemodialysis with low GA levels had better survival than those with high GA levels, regardless of whether the patients had diabetes. Thus, high GA levels are clearly a strong risk factor for the development of cardiovascular disease and death in patients undergoing hemodialysis.

The current study indicated that the GA levels in patients undergoing hemodialysis were associated with sudden death. Even after multivariate adjustment, the results indicated that a high GA level (≥18.5%) was a significant risk factor for the occurrence of sudden death. This trend was particularly strong among men aged <65 years.

GA plays a vital role in the physiological mechanism of diabetic atherosclerosis. It is converted into an advanced glycation end product under hyperglycemic conditions, and it is a key factor in the development of macrovascular complications of diabetes. Advanced glycation end products have been reported to precede plaque formation and large and small vessel atherosclerosis caused by the inflammatory response of vascular endothelial cells [12]. GA causes functional destruction of endothelial cells and induces oxidative stress, inflammatory responses in the vascular wall, and proliferation and migration of vascular smooth muscle cells, thus accelerating the development and progression of atherosclerosis and vascular complications [13]. The relationship between high GA levels and sudden death may involve a mechanism whereby GA causes the progression of atherosclerosis, leading to sudden cardiac death via lethal arrhythmias secondary to coronary artery disease.

According to the guidelines for the management of diabetic patients undergoing hemodialysis established by the Japan Society for Dialysis Therapy, the target GA level is <20% (tentatively) and the target casual blood glucose level is <180–200 mg/dL [14]. If the patient has a history of cardiovascular events and a tendency to develop hypoglycemia, the target GA level is <24%.

This study had four main limitations. First, several parameters that may influence the occurrence of sudden death were not evaluated, including interdialytic weight gain, the QRS duration, blood pressure variability during dialysis, the left ventricular ejection fraction, the left ventricular myocardial mass index, the presence or absence of valvular disease or vascular calcification, the brain natriuretic level, the presence or absence of sleep apnea, the presence or absence of atrial fibrillation, and whether patients were receiving medications such as an active form of vitamin D, erythropoietin, iron, or antihypertensives. Second, this was a single-center study with a small sample. Although there was a potential for bias in the analysis, the results did not change when the data were examined by sex and age. Despite these limitations, this study is meaningful because it indicates that GA levels may be associated with sudden death in patients undergoing dialysis.

Third, in this analysis, patients with diseases that affect GA levels, such as cirrhosis, thyroid disease and nephrotic syndrome were not excluded. However, GA levels tends to be low in these diseases, so it is unlikely to distort the present results. Forth, although hypoglycemia is also one of a risk of sudden death[15], it was very difficult to measure daily blood glucose fluctuations in all dialysis patients. Furthermore, we have no data on oral hypoglycemic drug or insulin users.

Patients undergoing dialysis have a high frequency of sudden death. The fact that the risk of sudden death was high even with a GA level of <20% in the current study suggests that GA may need to be controlled to a lower level to protect these patients from sudden death. From the perspective of preventing sudden death, GA may need to be maintained at an even lower level.

Further large-scale epidemiological studies are required to establish a target GA concentration that prevent sudden death for patients undergoing hemodialysis.

## Conclusions

The current study indicates that the frequency of sudden death is high in patients undergoing hemodialysis and that the GA level is associated with sudden death.

Further studies focusing on glycemic control are required to further ascertain the effects of GA on sudden death in patients undergoing dialysis.

### Supplementary Information

Below is the link to the electronic supplementary material.

Supplementary file1 (DOCX 48 KB)
