# Supplementary material for: Association between glycated albumin and sudden death in patients undergoing hemodialysis
Source: Clin Exp Nephrol. 2024 Mar 4;28(7):656–63. doi: 10.1007/s10157-024-02475-w (PMC11190002; doi:10.1007/s10157-024-02475-w)
Supplement: Supplementary file 1 — Supplementary file1 (DOCX 48 KB) [file 10157_2024_2475_MOESM1_ESM.docx]

| **Supplementary Table 3-a. Odds ratio for sudden death according to serum glycated albumin** | | | | | | | |
| --- | --- | --- | --- | --- | --- | --- | --- |
|  |  |  |  |  |  |  |  |
|  |  |  |  | Model 1^a^ |  | Model 2^b^ |  |
|  |  | Number  of events | Number  of subjects | OR (95%CI) | p-value | OR (95%C.I) | p-value |
|  |  |  |  |  |  |  |  |
| Glycated albumin | |  |  |  |  |  |  |
| T1 | <15.2 | 3 | 87 | 1.00 (reference) |  | 1.00 (reference) | - |
|  |  |  |  |  |  |  |  |
| T2 | 15.2-18.5 | 6 | 86 | 2.02 (0.45-9.04) | 0.36 | 1.44 (0.30-7.00) | 0.65 |
|  |  |  |  |  |  |  |  |
| T3 | ≥18.5 | 14 | 87 | 5.40 (1.35-21.85) | 0.002 | 2.50 (0.45-13.80) | 0.30 |
|  |  |  |  |  |  |  |  |
| ^a^Model 1: age and sex | | | | | | | |
| ^b^Model 2: age, sex, serum albumin level, serum C-reactive protein(CRP)level, and diabetes mellitus (DM) | | | | | | | |
| Abbreviations: OR, odds ratio; CI, confidence interval. | | | | | | | |

| **Supplementary Table 3-b. Odds ratio for sudden death according to serum glycated albumin** | | | | | | | |
| --- | --- | --- | --- | --- | --- | --- | --- |
|  |  |  |  |  |  |  |  |
|  |  |  |  | Model 1^a^ |  | Model 2^b^ |  |
|  |  | Number of events | Number of subjects | OR (95%CI) | p-value | OR (95%C.I) | p-value |
|  |  |  |  |  |  |  |  |
| Glycated albumin | |  |  |  |  |  |  |
| T1 | <15.2 | 3 | 87 | 1.00 (reference) |  | 1.00 (reference) | - |
|  |  |  |  |  |  |  |  |
| T2 | 15.2-18.5 | 6 | 86 | 2.02 (0.45-9.04) | 0.36 | 2.00 (0.43-9.18) | 0.38 |
|  |  |  |  |  |  |  |  |
| T3 | ≥18.5 | 14 | 87 | 5.40 (1.35-21.85) | 0.002 | 5.36 (1.32-21.72) | 0.02 |
|  |  |  |  |  |  |  |  |
| ^a^Model 1: age and sex | | | | | | | |
| ^b^Model 2: age, sex, serum albumin level, serum C-reactive protein (CRP) level, and history of CVD | | | | | | | |
| Abbreviations: OR, odds ratio; CI, confidence interval. | | | | | | | |

| **Supplementary Table3-c. Odds ratio for sudden death according to serum glycated albumin** | | | | | | | | |
| --- | --- | --- | --- | --- | --- | --- | --- | --- |
|  |  |  |  |  |  |  |  |  |
|  |  |  |  |  | Model 1^a^ |  | Model 2^b^ |  |
|  |  |  | Number of events | Number of subjects | OR (95%CI) | p-value | OR (95%C.I) | p-value |
|  |  |  |  |  |  |  |  |  |
| Glycated albumin | |  |  |  |  |  |  |  |
| T1 | <15.2 |  | 3 | 87 | 1.00 (reference) |  | 1.00 (reference) | - |
|  |  |  |  |  |  |  |  |  |
| T2 | 15.2-18.5 |  | 6 | 86 | 2.02 (0.45-9.04) | 0.36 | 2.53 (0.55-11.6) | 0.23 |
|  |  |  |  |  |  |  |  |  |
| T3 | ≥18.5 |  | 14 | 87 | 5.40 (1.35-21.85) | 0.002 | 5.50 (1.36-22.20) | 0.02 |
|  |  |  |  |  |  |  |  |  |
| ^a^Model 1: age and sex | | | | | | | | |
| ^b^Model 2: age, sex, serum, albumin level, serum C-reactive protein (CRP) level, and serum phosphate level | | | | | | | | |
| Abbreviations: OR, odds ratio; CI, confidence interval. | | | | | | | | |

| **Supplementary Table3-d. Odds ratio for sudden death according to serum glycated albumin** | | | | | |  |  |
| --- | --- | --- | --- | --- | --- | --- | --- |
|  |  |  |  |  |  |  |  |
|  |  |  |  | Model 1^a^ |  | Model 2^b^ |  |
|  |  | Number of events | Number  of subjects | OR (95%CI) | p-value | OR (95%C.I) | p-value |
|  |  |  |  |  |  |  |  |
| Glycated albumin | |  |  |  |  |  |  |
| T1 | <15.2 | 3 | 87 | 1.00 (reference) |  | 1.00 (reference) | - |
|  |  |  |  |  |  |  |  |
| T2 | 15.2-18.5 | 6 | 86 | 2.02 (0.45-9.04) | 0.36 | 1.90 (0.39-9.30) | 0.41 |
|  |  |  |  |  |  |  |  |
| T3 | ≥18.5 | 14 | 87 | 5.40 (1.35-21.85) | 0.002 | 2.80 (0.47-16.30) | 0.26 |
|  |  |  |  |  |  |  |  |
| ^a^Model 1: age and sex | | | | | | | |
| ^b^Model 2: age, sex, serum albumin level, serum C-reactive protein(CRP) level, diabetes mellitus, history of CVD, and serum phosphate level | | | | | | | |
| Abbreviations: OR, odds ratio; CI, confidence interval. | | | | | | | |

| **Supplementary Table 3-e. Odds ratio for sudden death according to serum glycated albumin** | | | | | | | |  |
| --- | --- | --- | --- | --- | --- | --- | --- | --- |
|  |  |  |  |  |  |  |  |  |
|  |  |  |  | Model 1^a^ |  |  | Model 2^b^ |  |
|  |  | Number of events | Number  of subjects | OR (95%CI) | p-value |  | OR (95%C.I) | p-value |
|  |  |  |  |  |  |  |  |  |
| Glycated albumin | |  |  |  |  |  |  |  |
| Q1 | <14.5 | 1 | 62 | 1.00 (reference) |  |  | 1.00 (reference) | - |
|  |  |  |  |  |  |  |  |  |
| Q2 | 14.5-16.6 | 4 | 63 | 4.42 (0.45-43.40) | 0.2 |  | 4.10 (0.37-44.44) | 0.24 |
|  |  |  |  |  |  |  |  |  |
| Q3 | 16.7-19.6 | 5 | 58 | 6.35 (0.65-61.50) | 0.11 |  | 7.83 (0.76-80.51) | 0.08 |
|  |  |  |  |  |  |  |  |  |
| Q4 | ≥19.6 | 13 | 54 | 16.72 (1.87-149.52) | 0.01 |  | 24.62 (2.64-230.00) | 0.005 |
|  |  |  |  |  |  |  |  |  |
| ^a^Model 1: age and sex | | | | | | | | |
| ^b^Model 2: age, sex, serum albumin level, serum C-reactive protein(CRP) level and cardiothoracic ratio(CTR) | | | | | | | | |
| Abbreviations: OR, odds ratio; CI, confidence interval. | | | | | | | | |

| **Table 3-f. Odds ratio for sudden death according to serum glycated albumin** | | | | | | | |  |
| --- | --- | --- | --- | --- | --- | --- | --- | --- |
|  |  |  |  |  |  |  |  |  |
|  |  |  |  |  | Model 1^a^ |  | Model 2^b^ |  |
|  |  |  | Number of events | Number  of subjects | OR (95%CI) | p-value | OR (95%C.I) | p-value |
|  |  |  |  |  |  |  |  |  |
| Glycated albumin | |  |  |  |  |  |  |  |
| Q1 | <14.2 |  | 1 | 52 | 1.00 (reference) | - | 1.00 (reference) | - |
|  |  |  |  |  |  |  |  |  |
| Q2 | 14.2-15.8 |  | 3 | 52 | 3.78 (0.36-40.3) | 0.27 | 2.96 (0.23-38.14) | 0.41 |
|  |  |  |  |  |  |  |  |  |
| Q3 | 15.9-17.5 |  | 3 | 53 | 3.73 (0.33-42.00) | 0.29 | 5.01 (0.41-61.71) | 0.21 |
|  |  |  |  |  |  |  |  |  |
| Q4 | 17.6-20.7 |  | 5 | 54 | 6.93 (0.68-70.40) | 0.10 | 8.35 (0.80-91.73) | 0.08 |
|  |  |  |  |  |  |  |  |  |
| Q5 | ≥20.8 |  | 11 | 49 | 17.34 (1.81-165.89) | 0.01 | 30.21 (2.85-320.27) | 0.005 |
|  |  |  |  |  |  |  |  |  |
| ^a^Model 1: age and sex | | | | | | | | |
| ^b^Model 2: age, sex, serum albumin level, serum C-reactive protein (CRP) level and cardiothoracic ratio (CTR) | | | | | | | | |
| Abbreviations: OR, odds ratio; CI, confidence interval. | | | | | | | | |

| **Table 4-a.Odds ratio for sudden death according to serum glycated albumin of PAD and non-PAD group** | | | | | | | | | | |
| --- | --- | --- | --- | --- | --- | --- | --- | --- | --- | --- |
| Glycated albumin | |  |  | |  | | Model 1^a^ |  | Model 2^b^ |  |
|  |  |  | Number | Number | | OR (95%CI) | | p-value | OR (95%C.I) | p-value |
|  |  |  | of events | of subjects | |  | |  |  |  |
| PAD | |  |  |  | |  | |  |  |  |
| T1 | <15.2 |  | 0 | 6 | | 1.00 (reference) | |  | 1.00 (reference) | - |
|  |  |  |  |  |  | |  |  |  |  |
| T2 | 15.2-18.5 |  | 0 | 13 | | - | |  | - | - |
|  |  |  |  |  |  | |  |  |  |  |
| T3 | ≥18.5 |  | 5 | 31 | | - | |  | - | - |
| non-PAD | |  |  |  | |  | |  |  |  |
| T1 | <15.2 |  | 3 | 81 | | 1.00 (reference) | |  | 1.00 (reference) | - |
|  |  |  |  |  |  | |  |  |  |  |
| T2 | 15.2-18.5 |  | 6 | 73 | | 2.0 (0.40-9.20) | | 0.38 | 2.2 (0.41-11.60) | 0.36 |
|  |  |  |  |  |  | |  |  |  |  |
| T3 | ≥18.5 |  | 9 | 56 | | 4.40(0.99-19.10) | | 0.05 | 5.50 (1.20-25.50) | 0.03 |
| ^a^Model 1: age and sex | | | | | | | | | | |
| ^b^Model 2: age, sex, serum albumin level, serum C-reactive protein (CRP) level, and cardiothoracic ratio (CTR) | | | | | | | | | | |
| Abbreviations: OR, odds ratio; CI, confidence interval  . | | | | | | | | | | |

| **Supplementary Table 4-b. Odds ratio for sudden death according to serum glycated albumin of DM**  **and non-DM** | | | | | | | | | |
| --- | --- | --- | --- | --- | --- | --- | --- | --- | --- |
| Glycated albumin | |  |  | |  | Model 1^a^ |  | Model 2^b^ |  |
|  |  |  | Number | Number | OR (95%CI) | | p-value | OR (95%C.I) | p-value |
|  |  |  | of events | of subjects |  | |  |  |  |
| DM | |  |  |  |  | |  |  |  |
| T1 | <15.2 |  | 0 | 4 | 1.00 (reference) | |  | 1.00 (reference) | - |
|  |  |  |  |  |  |  |  |  |  |
| T2 | 15.2-18.5 |  | 2 | 19 | - | | - | - | - |
|  |  |  |  |  |  |  |  |  |  |
| T3 | ≥18.5 |  | 13 | 63 | - | | - | - | - |
| non-DM | |  |  |  |  | |  |  |  |
| T1 | <15.2 |  | 3 | 83 | 1.00 (reference) | |  | 1.00 (reference) | - |
|  |  |  |  |  |  |  |  |  |  |
| T2 | 15.2-18.5 |  | 4 | 67 | 1.40 (0.24-7.80) | | 0.70 | 1.20 (0.12-11.40) | 0.90 |
|  |  |  |  |  |  |  |  |  |  |
| T3 | ≥18.5 |  | 1 | 24 | 1.32 (0.11-16.50) | | 0.80 | 1.75 (0.11-28.70) | 0.70 |
| ^a^Model 1: age and sex | | | | | | | | | |
| ^b^Model 2: age, sex, serum albumin level, serum C-reactive protein (CRP) level and cardiothoracic ratio (CTR) | | | | | | | | | |
| Abbreviations: OR, odds ratio; CI, confidence interval  . | | | | | | | | | |
